# A dual RPA-LFD assay for the simultaneous detection of *Salmonella typhimurium* and *Salmonella enteritidis*

**DOI:** 10.3389/fbioe.2024.1379939

**Published:** 2024-03-08

**Authors:** Chuan Liao, Lele Pan, Meiying Tan, Zihan Zhou, Shaoping Long, Xueli Yi, Xuebin Li, Guijiang Wei, Lina Liang

**Affiliations:** ^1^ Center for Medical Laboratory Science, Affiliated Hospital of Youjiang Medical University for Nationalities, Baise, China; ^2^ Baise Key Laboratory for Research and Development on Clinical Molecular Diagnosis for High-Incidence Diseases, Baise, China; ^3^ Key Laboratory of Research on Clinical Molecular Diagnosis for High Incidence Diseases in Western Guangxi, Baise, China; ^4^ Department of Clinical Laboratory, Baise People’s Hospital, Baise, China; ^5^ Department of Neurology, Affiliated Hospital of Youjiang Medical University for Nationalities, Baise, China; ^6^ Modern Industrial College of Biomedicine and Great Health, Youjiang Medical University for Nationalities, Baise, China

**Keywords:** *Salmonella typhimurium*, *Salmonella* enteritidis, dual recombinase polymerase amplification, lateral flow dipstick, simultaneous detection

## Abstract

**Introduction:** *Salmonella* was one of the most common bacteria that caused foodborne illness, with *S. typhimurium* (*Salmonella typhimurium*) and *S. enteritidis* (*Salmonella enteritidis*) infections accounting for more than 75% of human salmonella infections.

**Methods:** In this study, we developed a method of dual recombinase polymerase amplification (RPA) combined with a lateral flow dipstick for the rapid detection of *S. typhimurium* and *S. enteritidis* in clinical specimens (stool).

**Results:** The entire reaction process, including amplification and result reading, could be completed within 65 min. The detection limits of *S. typhimurium* and *S. enteritidis* in pure culture samples were 5.23 × 10^1^ CFU/mL and 3.59 × 10^1^ CFU/mL, respectively. The detection limits of *S. typhimurium* and *S. enteritidis* in artificially contaminated samples were 8.30 × 10^1^ CFU/mL and 2.70 × 10^2^ CFU/mL, respectively. In addition, the method had no cross-reaction with other pathogenic microorganisms. The results in clinical samples were fully consistent with those obtained using Bacterial Analysis Manual, with sensitivity and specificity were 100% (8/8) and 100% (17/17) for *S. typhimurium* and 100% (4/4) and 100% (21/21) for *S. enteritidis*, respectively.

**Discussion:** The detection limits of *S. typhimurium* and *S. enteritidis* in artificially contaminated samples were higher than those in pure culture samples, which might be attributed to the inherent complex composition of artificially contaminated samples. In addition, the detection limits of *S. typhimurium* and *S. enteritidis* in the same sample were also different, which might be attributed to different amplification efficiency of two target genes in the same reaction system.

**Conclusion:** This assay had potential application outdoors, as it could be performed within 1 h at 38°C without a complex instrument, and the results could be observed with the naked eye. In conclusion, the dual RPA-LFD assay established in this study had practical significance for the rapid detection of *S. typhimurium* and *S. enteritidis* in the future.

## 1 Introduction

*Salmonella* was a group of rod-shaped gram-negative facultative anaerobic bacteria in the Enterobacteriaceae family (Brown et al., 2021). *Salmonella* was widely distributed in the natural environment and often persisted in other natural environments outside of animals, including fresh and marine surface water, and as epiphytes on and inside plant material (Bell et al., 2015). To date, there are more than 8 million cases of foodborne salmonellosis worldwide each year, it is one of the most important foodborne pathogens worldwide (Ha et al., 2021). *Salmonella* serotypes had more than 2,500 (Takaya et al., 2020), in the world’s bacterial food poisoning cases, its food poisoning often ranked first or second (Golden and Mishra, 2020). A survey in the UK showed that *S. typhimurium* (*Salmonella typhimurium*) and *S. enteritidis* (*Salmonella enteritidis*) infections accounted for more than 75% of human *Salmonella* infections (Abukhattab et al., 2022; Ferrari et al., 2019). Food containing *Salmonella* bacteria could lead to food poisoning, even gastroenteritis, septicemia, and other symptoms (Abdelhaseib et al., 2016; Bell et al., 2015). As a result, the World Health Organization classified it as a foodborne pathogen with moderate to serious harm.

Intestinal diseases caused by *Salmonella* bacteria are usually caused by cross-contamination of food with the feces of infected animals, or by contamination from the environment or other food sources, so timely detection of the bacteria was key to control it (Rogers et al., 2021; Sahu et al., 2019; Thomas-Lopez et al., 2018). At present, *Salmonella* was mainly detected by traditional laboratory methods. These traditional laboratory methods were cumbersome and time-consuming (Wang et al., 2021; Xu et al., 2019). In recent years, with the continuous development and improvement of nucleic acid detection technologies, many detection methods based on nucleic acid amplification have become popular, such as polymerase chain reaction (PCR) and loop-mediated isothermal amplification (LAMP) (Lai et al., 2024). Although the PCR method had high sensitivity and specificity, it required trained personnel and complex thermal cyclers, which made it difficult to be used in under-equipped laboratories and low-resource field environments (Zhu et al., 2020). Although the LAMP method was short in time and got rid of the dependence on the instrument, it had high false positives and was easy to be contaminated (Alhamid et al., 2023).

Recombinase polymerase amplification (RPA) was one of the isothermal amplification technologies. RPA had the advantage of completing amplification within 30 min at relatively low temperatures (37°C–42 °C) (Piepenburg et al., 2006). At the temperature of 37°C–42 °C, recombinant enzyme *uvsX* bound closely with the primer to form a complex. When the primer found a completely complementary sequence on the template DNA, the complex divided the template DNA, and a new complementary DNA chain was formed by strand replacement DNA polymerase Bsu. In this system, the DNA amplification process was very fast, generally within 30 min to reach a detectable amount of amplified products (Piepenburg et al., 2006). RPA-based methods were successful in detecting some pathogenic microorganisms (Choi et al., 2023; Dao et al., 2018). However, the simultaneous detection of *S. typhimurium* and *S. enteritidis* in clinical samples had not been reported.

In this study, the STM4497 gene of *S. typhimurium* and the safA gene of *S. enteritidis* were chosen as detected targets, and 5 pairs of primers were designed. After optimizing the amplification conditions of RPA, a dual RPA amplification method combined with lateral flow dipstick (LFD) as signal reading method (dual RPA-LFD assay) was successfully established. Compared to previous methods that just detected *S. typhimurium* or *S. enteritidis* at one time, the dual RPA-LFD assay was a novel multiple nucleic acid amplification method for the simultaneous detection of *S. typhimurium* and *S. enteritidis,* and has not been reported before. It not only maintained the specificity and sensitivity of single-target nucleic acid detection but also reduced the number of procedures and reagents, and was hoped to provide a novel and promising method for the simultaneous detection of *S. typhimurium* and *S. enteritidis*.

## 2 Experimental section

### 2.1 Bacterial strains and DNA template preparation

The standard strains used in this study are listed in Table 1. These strains were employed to assess the specificity of the dual RPA-LFD assay. For this purpose, *S. typhimurium* and *S. enteritidis* were utilized as reference strains. Using these strains, the reaction system of the dual RPA-LFD assay was optimized, along with its detection limit analysis. The above strains were cultured in the Luria-Bertani (LB) medium at 37 °C for 24 h. Bacterial cultures were used for nucleic acid extraction or routine plate counting. To determine the detection limit, *S. typhimurium* and *S. enteritidis* solution were serially diluted 10 fold, then 1 mL of these diluted solutions were added into LB medium and incubated at 37 °C for 24 h. To avoid laboratory personnel infection and sample contamination, the whole operation process must be conducted in the biosafety cabinet, and the LB medium culture dishes were inverted during culturing. According to the Bacterial Analysis Manual (BAM), the number of bacterial colonies per plate (repeated 3 times) multiplied by the dilution ratio was the number of bacteria per milliliter (CFU/mL). Notably, only plates containing 30 to 300 colonies could be used for plate counting.

**TABLE 1:** Information of bacterial strains used for specificity tests.

Species	ID of strains	Dual RPA-LFD assay	
		STM4497	safA
*S. typhimurium*	ATCC14028	+	-
*S. enteritidis*	ATCC13076	-	+
*K. pneumoniae*	ATCC700603	-	-
*E. coli*	ATCC25922	-	-
*F. faecalis*	ATCC35667	-	-

*Salmonella typhimurium* (*S. typhimurium*), *Salmonella* enteritidis (S. enteritidis), *Klebsiella pneumoniae* (*K. pneumoniae*), *Escherichia coli* (*E. coli*), and *Enterococcus faecalis* (*E. faecalis*).

The DNA of the bacteria listed in Table 1 was extracted according to the instructions of the bacterial DNA extraction kit (TIANGEN, Beijing). After centrifugation of the bacterial solution, the cell wall was removed by lysozyme digestion. The DNA was released from the cell through lysate lysis and protease K digestion. The DNA was added to the binding solution to adjust the optimal binding conditions. The DNA solution was transferred to the purification column and centrifuged. The DNA was selectively bound to the filter membrane, while impurities such as proteins entered the filtrate. The residual contaminants and enzyme inhibitors were removed through two washing steps. Finally, the DNA was eluted with a small amount of buffer and stored at −80 °C.

### 2.2 Design of the RPA primers

Nucleic acid sequences of the STM4497 gene of *S. typhimurium* and the safA gene of *S. enteritidis* were obtained from NCBI. The RPA primers were designed using Primer Premier 5.0 software according to the guidelines provided by the TwistAmp™Basic Kit (TwistDx, US). Specific parameters were set as follows: Primer pair length was between 32–35 bp, GC content was between 30% and 70%, amplicon length was between 100–300 bp, Tm value was between 50% and 100%, and a maximum allowable length of a mononucleotide repeat was “5". Additionally, primer specificity was initially confirmed by homologous comparison with available sequences on NCBI. Subsequently, experimental analysis was conducted to select the primers with the highest amplification efficiency. Furthermore, their specificity was verified by amplifying DNA from various bacterial strains. The best-selected primer pairs (Table 2) were labeled with biotin and digoxin, carboxyfluorescein (FAM), and digoxin. All primers were synthesized by Shanghai Shenggong Bioengineering Technology Service Corporation.

**TABLE 2:** The sequences of RPA primers.

Primer name	Sequence (5′–3′)	Primer length (bp)
*ST*-F1	5′-GTG​CTT​GAA​TAC​CGC​CTG​TCA​CAG​GTT​CAG​AGC-3′	177
*ST*-R1	5′-CTT​GTG​GTC​CTT​TTC​CAG​ATT​ACG​CAA​CAG​ATA-3′	
*ST*-F2	5′-CTG​ACA​GGA​ATG​GGT​AAC​GCC​TGG​CCG​CTG​GTT-3′	139
*ST*-R2	5′-CCG​CCA​ATG​GGG​AGA​GAT​CGT​GTC​CGC​TAT​AGG-3′	
*ST*-F3	5′-GTG​CTT​GAA​TAC​CGC​CTG​TCA​CAG​GTT​CAG​AGC-3′	189
*ST*-R3	5′-GAG​GTG​CGC​GAA​CTT​GTG​GTC​CTT​TTC​CAG​ATT-3′	
*ST*-F4	5′-Biotin-GTGCTTGAATACCGCCTGTCACAGGTTCAGAGC-3′	187
*ST*-R4	5′-Digoxin-GGTGCGCGAACTTGTGGTCCTTTTCCAGATTAC-3′	
*ST*-F5	5′-TTG​AAT​ACC​GCC​TGT​CAC​AGG​TTC​AGA​GCC​GCA-3′	169
*ST*-R5	5′-TGG​TCC​TTT​TCC​AGA​TTA​CGC​AAC​AGA​TAC​TTC-3′	
*SE*-F1	5′-TAA​ATG​TGT​TTT​ATC​TGA​TGC​AAG​AGG​GGG​AG-3′	228
*SE*-R1	5′-GAT​TTA​CTA​AAC​TTT​TTG​ATA​TAC​TCC​CTG​AAT​C-3′	
*SE*-F2	5′-CGA​CCG​GAT​TTG​GCC​GAT​CTA​ATG​AAC​TAC​GT-3′	219
*SE*-R2	5′-TTG​TGC​AGC​GAG​CAT​GTT​CTG​GAA​AGC​CTC​TT-3′	
*SE*-F3	5′-CGT​CAG​TCA​CTA​TTT​GAA​CGG​TAA​GGC​ACC​TC-3′	270
*SE*-R3	5′-TCA​TTC​TGA​CCT​TTA​AGC​CGG​TCA​ATG​AGT​TT-3′	
*SE*-F4	5′-FAM-TATCGCTTATTCCAGTGTCGGGCTTAAAGGCT-3′	262
*SE*-R4	5′-Digoxin-TTAAAGGTCATCCAGTTCACCCCATTCCATTT-3′	
*SE*-F5	5′-ATC​GGT​CCT​GCT​GTA​GAT​GCA​AGG​GTG​CCT​AA-3′	268
*SE*-R5	5′-CTC​CAC​TTG​GTT​CAA​ACC​TCG​CCC​TCA​CAT​TC-3′	

*Salmonella typhimurium* (ST) and *Salmonella* enteritidis (SE).

### 2.3 Dual RPA reactions in solution

The dual RPA amplification system consisted of 29.5 μL primerless rehydration buffer, 1.2 μL forward and 1.2 μL reverse primer of *S. typhimurium*, 1.2 μL forward and 1.2 μL reverse primer of *S. enteritidis*, 2.5 μL magnesium acetate, 13.2 μL DNA template, and water. The steps of the dual RPA amplification system were as follows: the components without template DNA were fully mixed; then the mixture was added to a reaction tube containing lyophilized enzyme powder and mixed gently with a pipette; two kinds of template DNA were added to the test tube, and 2.5 μL magnesium acetate was added to the test tube lid. After centrifugation, the reaction was performed under a constant temperature for 20 min. Finally, the amplification products of RPA were detected by LFD and gel electrophoresis.

### 2.4 The design of LFD

**FIGURE 1:**
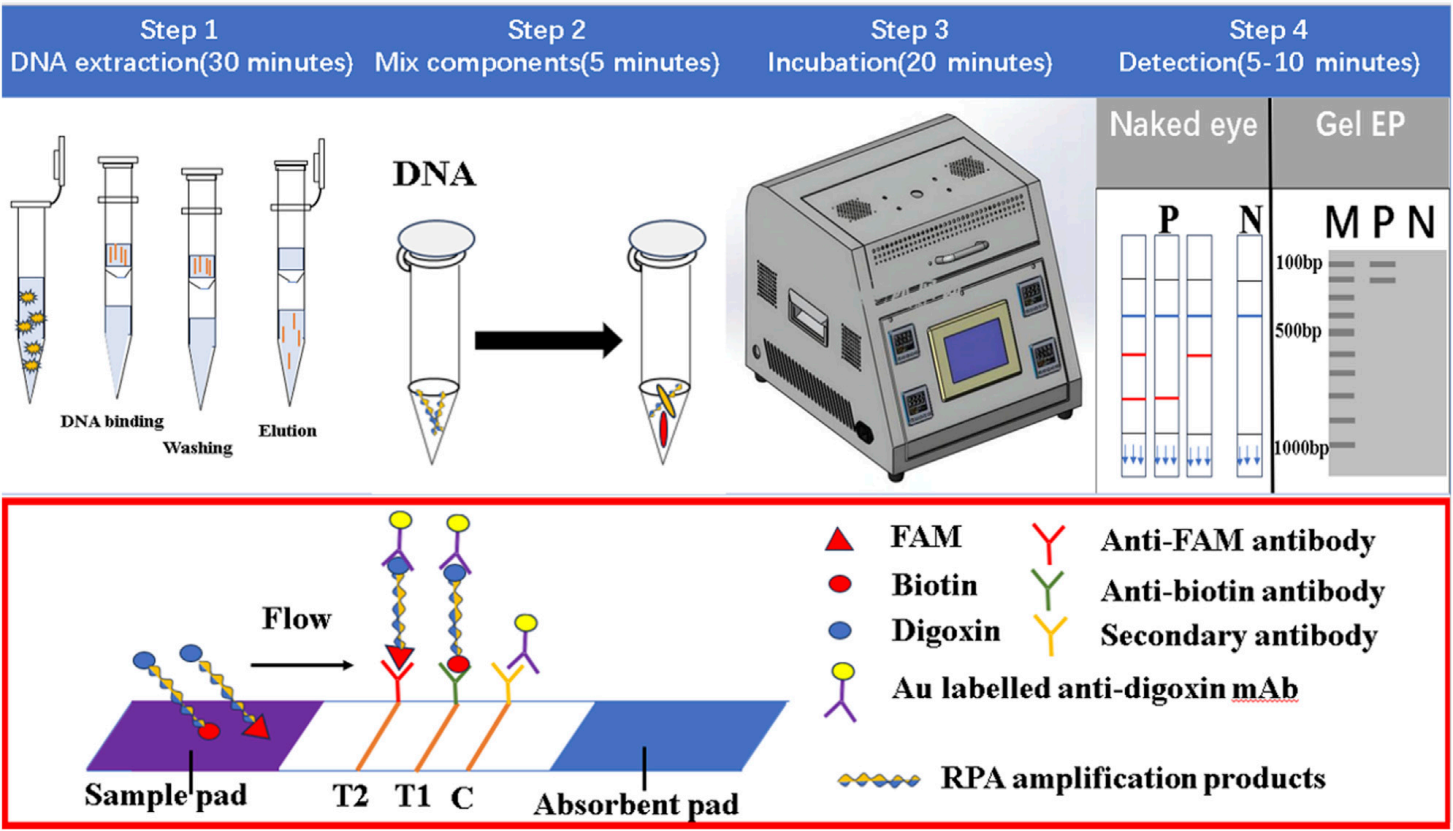
The working principle of dual RPA-LFD assay. **Step 1:** The DNA of *ST* and *SE* was extracted using a DNA extraction kit. **Step 2 and 3:** The dual RPA amplification was performed using RPA primers to produce biotin and digoxin, or FAM and digoxin-labeled DNA amplification products. **Step 4:** The amplification products were diluted 50 times and added to the sample pad, when the amplified solution migrated to the other end, the labeled DNA products were bound to the Au-labeled anti-digoxin monoclonal antibody on the binding pad. When the amplification solution continued to migrate to the test line, biotin or FAM-labeled DNA products were captured on the T1 (anti-biotin monoclonal antibodies) or T2 (anti-FAM monoclonal antibodies) test line. The excess Au-labeled anti-digoxin monoclonal antibodies were captured by the control line (secondary antibody). In the absence of target DNA, red bands did not appear on the T1 or T2 test line. If RPA amplification was successful, red bands would be produced on the test and control lines. *Salmonella typhimurium* (*ST*), *Salmonella enteritidis* (*SE*).

The LFD was mainly composed of a sample pad, binding pad, absorption pad, liner, and nitrocellulofiltration membrane, with two test lines and one control line (Figure 1). The Au-labeled anti-digoxin monoclonal antibody was sprayed on the binding mat, and then the two test lines T1 and T2 were prepared with anti-biotin monoclonal antibody and anti-FAM monoclonal antibody respectively. The fixed anti-digoxin anti-antibody on the control line was used as the assay control. Before pipetting the liquid onto the test strip, the amplification products were diluted 50–100 times with ultra-pure water. The result of the test line would be read after 5–10 min.

### 2.5 Dual RPA-LFD assay

The principle of dual RPA-LFD assay is shown in Figure 1. Firstly, the DNA of *S. typhimurium* and *S. enteritidis* was extracted using a DNA extraction kit. Then dual RPA amplification was performed using labeled upstream and downstream primers to produce biotin and digoxin, or FAM and digoxin labeled double-stranded DNA amplification products. Finally, the amplification products were diluted 50 times and added to the sample pad, and the results were observed after 5–10 min.

When the amplified solution migrated to the other end, the labeled double-stranded DNA products were bound to the Au-labeled anti-digoxin monoclonal antibody on the binding pad. When the amplification solution continued to migrate to the test line, biotin or FAM-labeled double-stranded DNA products were captured on the T1 or T2 test line containing corresponding anti-biotin monoclonal antibodies or anti-FAM monoclonal antibodies, respectively. The excess gold nanoparticles were captured by control lines immobilized with a secondary antibody. In the absence of target DNA, red bands did not appear on the T1 or T2 test line. If amplification was successful, red bands would be produced on the test and control lines. The entire detection process, including amplification and reading of results, needed about 30 min.

### 2.6 Optimization of the dual RPA-LFD assay conditions

To establish a dual RPA assay, preliminary single-tube experiments were performed to screen the optimal primer pair and primer ratio and to test different reaction conditions. The single-tube amplification system for *S. typhimurium* and *S. enteritidis* was optimized. The amplification efficiency of different targets was inconsistent in the experiment. Different concentration ratios of the two primers were investigated to determine the optimal primer ratio to achieve similar amplification efficiency of the two fragments. To determine the optimal ratio of STM4497 primers and safA primers, we set the ST-F4/R4: SE-F4/R4 ratio to 10 μM: 2 μM, 10 μM: 4 μM, 10 μM: 6 μM, 10 μM: 8 μM, 10 μM: 10 μM, 2 μM: 10 μM, 4 μM: 10 μM, 6 μM: 10 μM, and 8 μM: 10 μM, then added equal amounts of primers with different concentrations in reaction system, finally performed the reaction at 38 °C for 20 min. Gel electrophoresis analysis was used to detect the product bands and the optimal primer ratio was determined by observing the brightness of the product bands. Through the primer optimization, the optimal reaction temperature, reaction time, and DNA concentration of dual RPA were also investigated. The dual RPA reaction was performed at five different reaction temperatures (35 °C, 37 °C, 38 °C, 39 °C, and 41 °C), five different reaction times (20 min, 25 min, 30 min, 35 min, and 40 min), and three different template DNA concentrations (1 μL: 1 μL, 2 μL: 2 μL, and 3 μL: 3 μL). Gel electrophoresis analysis was used to detect the product bands and the optimal reaction temperature, reaction time, and DNA concentration were determined by observing the brightness of the product bands. Genomic DNA (10^7^ CFU/mL) of *S. typhimurium* and *S. enteritidis* were extracted as target templates according to the above protocol. All optimization experiments were carried out in a metal bath.

### 2.7 Detection limits and specificity of the dual RPA-LFD assay

In detection limit experiments, the detection limits were determined using a tenfold series dilution of two reference strains. Genomic DNA was extracted from bacterial cultures of *S. typhimurium* and *S. enteritidis* at concentrations ranging from 10^0^ to 10^7^ CFU/mL. Each concentration level was amplified three times, and the resulting products were subjected to gel electrophoresis and tested with strips for detection purposes. The genomic DNA (10^7^ CFU/mL) extracted from the five bacterial strains listed in Table 1 was incorporated into the reaction to ascertain the specificity of the dual RPA-LFD assay.

### 2.8 Application of the dual RPA-LFD assay in artificially contaminated samples

Both *S. typhimurium* and *S. enteritidis* were cultured in LB medium at 37°C for 18 h, and the cultures were utilized to prepare template DNA solution at various concentrations. Stool samples were collected from the Affiliated Hospital of Youjiang Medical University for Nationalities, all of which tested negative for *S. typhimurium* and *S. enteritidis* using the BAM method established by the US Food and Drug Administration. Subsequently, these collected samples were artificially contaminated with different concentrations of *S. typhimurium* and *S. enteritidis* ranging from 10^0^–10^7^ CFU/mL. Each sample was then subjected to DNA extraction.

To extract bacterial DNA from the artificially contaminated samples, 20 mg/mL protease K was added to digest the protein in the sample and destroy the bacterial cell wall. The DNA extracted from each sample was used in a dual RPA amplification reaction. Contaminated samples were also tested according to BAM. Uncontaminated samples were used as negative controls.

### 2.9 Clinical sample testing

To evaluate the clinical applicability, the dual RPA-LFD assay was employed for the analysis of 25 stool samples from the Affiliated Hospital of Youjiang Medical University for Nationalities. Among these, 8 stool samples tested positive for *S. typhimurium*, 4 were positive for *S. enteritidis*, and 13 were negative for both *S. typhimurium* and *S. enteritidis*. Untreated stool samples or extracted DNA were stored at −80°C. The samples underwent testing using two different methods: BAM and the dual RPA-LFD assay.

### 2.10 Data analysis

LFD and gel electrophoresis results were visually read. The optimum conditions were selected according to the brightness of the strips. Data collected from the dual RPA-LFD assay and culture methods were analyzed, including the positive detection rates of *S. typhimurium* and *S. enteritidis* in clinical samples with BAM and the dual RPA-LFD assay.

## 3 Results

### 3.1 Primer screening

**FIGURE 2:**
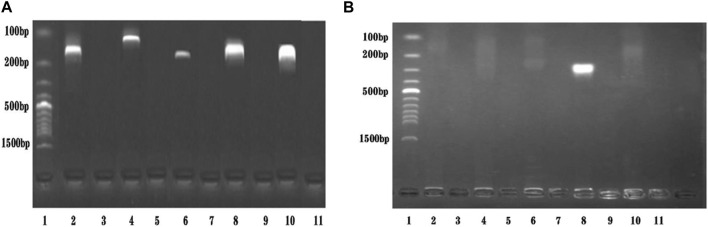
Primers screening. **(A).** Primers screening for *ST*: Lane 1 was DNA ladder; Lane 2, 4, 6, 8, and 10 were *ST*-F1/R1, *ST*-F2/R2, *ST*-F3/R3, *ST*-F4/R4, and *ST*-F5/R5, respectively; Lane 3, 5, 7, 9, and 11 were negative controls; The optimum primer pair was *ST*-F4/R4. **(B)**. Primers screening for *SE*: Lane 1 was DNA ladder; Lane 2, 4, 6, 8, and 10 were *SE*-F1/R1, *SE*-F2/R2, *SE*-F3/R3, *SE*-F4/R4, and *SE*-F5/R5, respectively; Lane 3, 5, 7, 9, and 11 were negative controls; The optimum primer pair was *SE*-F4/R4. *Salmonella typhimurium* (*ST*), *Salmonella enteritidis* (*SE*).

A primer in RPA required longer oligonucleotides, typically 30–35 base pairs. For the STM4497 gene of *S. typhimurium* and the safA gene of *S. enteritidis*, a total of 5 primer pairs were designed (Figure 2). Among these, F4/R4 primers exhibited the highest amplification efficiency with single bands. Consequently, this primer pair was selected for subsequent dual RPA-LFD assay experiments.

### 3.2 Optimization of dual RPA amplification reaction condition

**FIGURE 3:**
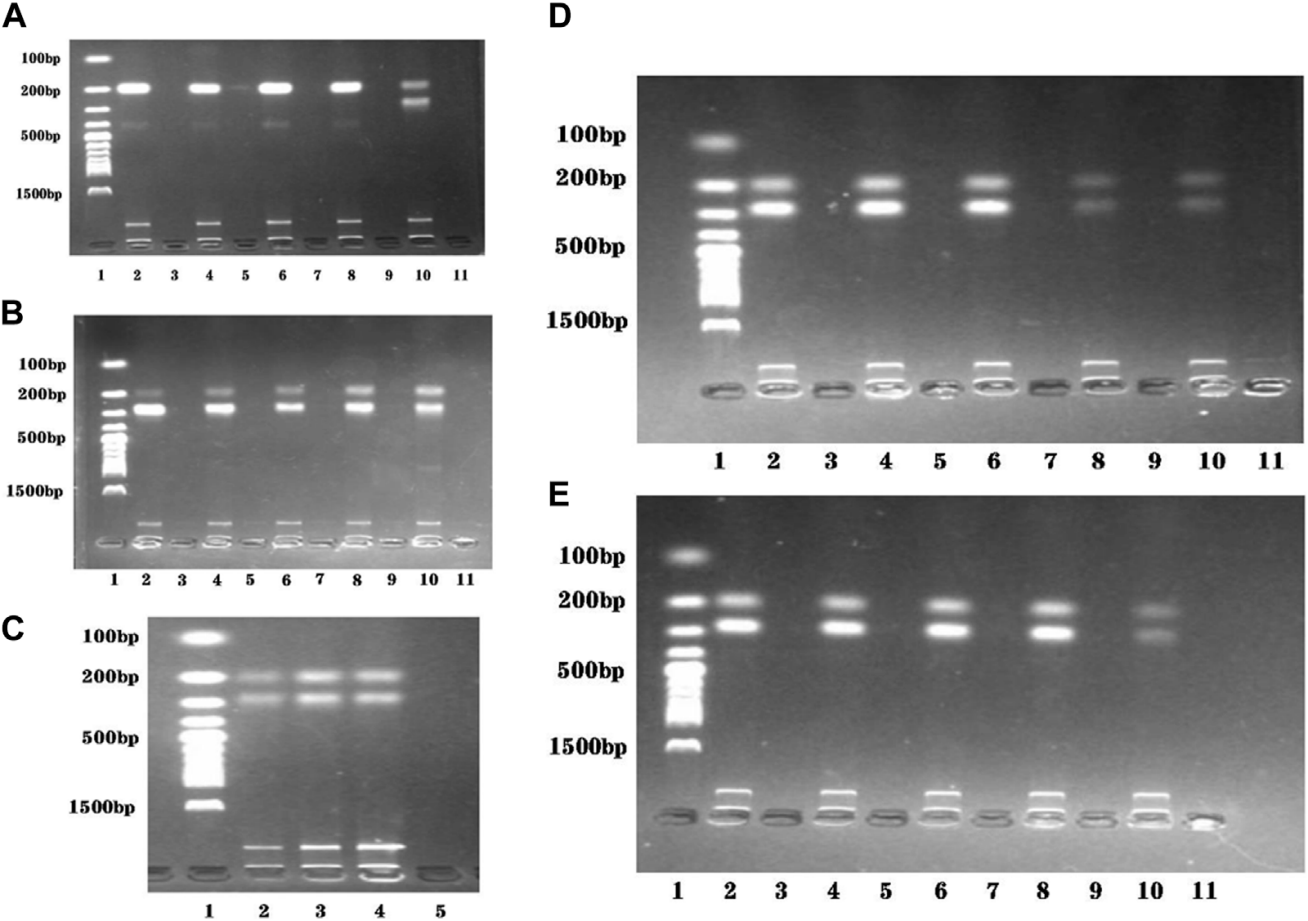
Reaction conditions optimization of the dual RPA-LFD assay. **(A).** The ratio optimization of *ST*-F4/R4: *SE*-F4/R4: Lane 1 was DNA ladder; Lane 2, 4, 6, 8, and 10 were 10 μM: 2 μM, 10 μM: 4 μM, 10 μM: 6 μM, 10 μM: 8 μM, and 10 μM: 10 μM, respectively; Lane 3, 5, 7, 9, and 11 were negative controls; The optimum ratio of *ST*-F4/R4: *SE*-F4/R4 was 10 μM: 10 μM. **(B).** The ratio optimization of *ST*-F4/R4: *SE*-F4/R4: Lane 1 was DNA ladder; Lane 2, 4, 6, 8, and 10 were 2 μM: 10 μM, 4 μM: 10 μM, 6 μM: 10 μM, 8 μM: 10 μM, and 10 μM: 10 μM, respectively; Lane 3, 5, 7, 9, and 11 were negative controls; The optimum ratio of *ST*-F4/R4: *SE*-F4/R4 was still 10 μM: 10 μM. **(C).** The target DNA concentration optimization (*ST* + *SE*): Lane 1 was DNA ladder; Lane 2, 3, and 4 were 1 μL + 1 μL, 2 μL + 2 μL, and 3 μL + 3 μL, respectively; Lane 5 was the negative control; The optimum target DNA concentrations (*ST* + *SE*) was 2 μL + 2 μL. **(D).** The amplification temperature optimization of dual RPA: Lane 1 was DNA ladder; Lane 2, 4, 6, 8, and 10 were 35 °C, 37 °C, 38 °C, 39 °C, and 41 °C, respectively; Lane 3, 5, 7, 9, and 11 were negative controls; The optimum amplification temperatures was 38 °C. **(E).** The amplification time optimization of dual RPA: Lane 1 was DNA ladder; Lane 2, 4, 6, 8, and 10 were 20 min, 25 min, 30 min, 35 min, and 40 min respectively; Lane 3, 5, 7, 9, and 11 were negative controls; The optimum amplification time was 20 min. *Salmonella typhimurium* (*ST*), *Salmonella enteritidis* (*SE*).

The presence of two primers in the dual RPA amplification reaction might result in mutual interference between them. To determine the optimal ratio of STM4497 primers and safA primers. We assessed gel electrophoresis bands after adding equal amounts of primers with different concentrations at 38 °C for 20 min (Figures 3A,B). When the ratio reached 1:1, minimal interference was observed and the amplification efficiency was maximized. Additionally, we investigated the impact of varying template amounts on amplification efficiency by adding different quantities of templates under identical conditions (Figure 3C). The highest amplification efficiency was achieved when adding 2 μL each of genomic DNA from *S. typhimurium* and *S. enteritidis*.

To evaluate the optimal amplification temperature, we incubated the dual RPA reaction system at different temperatures (35 °C, 37 °C, 38 °C, 39 °C, and 41 °C) in a metal bath for 30 min (Figure 3D). Gel electrophoresis analysis was used to detect the product bands. The brightest band was obtained at a temperature of 38 °C.

In order to determine the optimal reaction time, we performed the dual RPA amplification reaction at a constant temperature of 38°C for various durations (20 min, 25 min, 30 min, 35 min, and 40 min). Gel electrophoresis analysis was used to detect the product bands and the optimum reaction temperature was determined by observing the brightness of the product bands. As depicted in Figure 3E, the product band became visible after only 20 min. However, as time extended to 40 min, the product band did not become brighter. Therefore, we selected 20 min as the most suitable duration for performing the dual RPA amplification reaction.

### 3.3 Detection limits and specificity of the dual RPA-LFD assay

**FIGURE 4:**
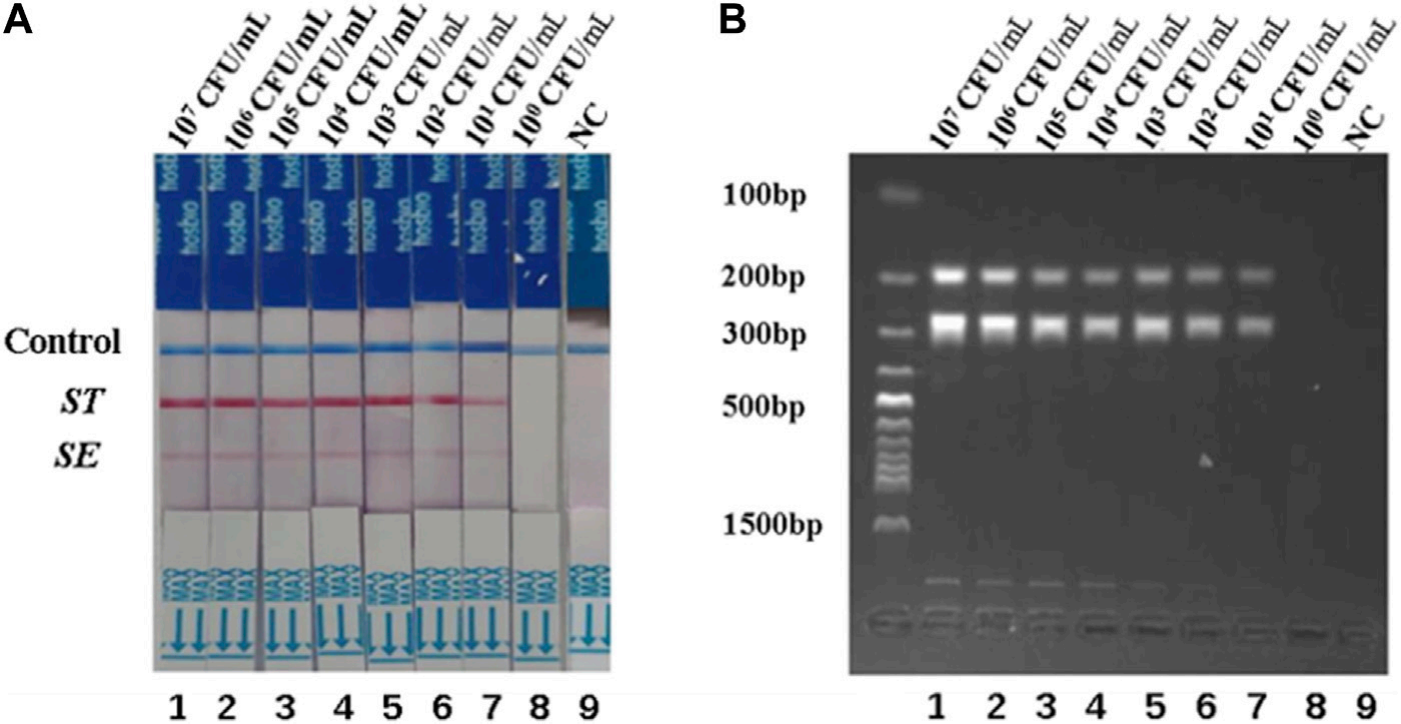
The detection limits of dual RPA-LFD assay and gel electrophoresis for *ST* and *SE* in pure bacteria samples. **(A).** The detection limit of dual RPA-LFD assay: Strip 1 (*ST*-5.23 × 10^7^ CFU/mL, *SE*-3.59 × 10^7^ CFU/mL), Strip 2 (*ST*-5.23 × 10^6^ CFU/mL, *SE*-3.59 × 10^6^ CFU/mL), Strip 3 (*ST*-5.23 × 10^5^ CFU/mL, *SE*-3.59 × 10^5^ CFU/mL), Strip 4 (*ST*-5.23 × 10^4^ CFU/mL, *SE*-3.59 × 10^4^ CFU/mL), Strip 5 (*ST*-5.23 × 10^3^ CFU/mL, *SE*-3.59 × 10^3^ CFU/mL), Strip 6 (*ST*-5.23 × 10^2^ CFU/mL, *SE*-3.59 × 10^2^ CFU/mL), Strip 7 (*ST*-5.23 × 10^1^ CFU/mL, *SE*-3.59 × 10^1^ CFU/mL), Strip 8 (*ST*-5.23 × 10^0^ CFU/mL, *SE*-3.59 × 10^0^ CFU/mL), Strip 9 (NC). When the bacterial concentrations of *ST* and *SE* were below 10^1^ CFU/mL, the color of the test line was not visible for the naked eye. Therefore, the detection limits of dual RPA-LFD assay for *ST* and *SE* in pure bacteria samples were 5.23×10^1^ and 3.59 × 10^1^ CFU/mL, respectively. **(B)**. The detection limit of gel electrophoresis: Lane 1 (*ST*-5.23 × 10^7^ CFU/mL, *SE*-3.59 × 10^7^ CFU/mL), Lane 2 (*ST*-5.23 × 10^6^ CFU/mL, *SE*-3.59 × 10^6^ CFU/mL), Lane 3 (*ST*-5.23 × 10^5^ CFU/mL, *SE*-3.59 × 10^5^ CFU/mL), Lane 4 (*ST*-5.23 × 10^4^ CFU/mL, *SE*-3.59 × 10^4^ CFU/mL), Lane 5 (*ST*-5.23 × 10^3^ CFU/mL, *SE*-3.59 × 10^3^ CFU/mL), Lane 6 (*ST*-5.23 × 10^2^ CFU/mL, *SE*-3.59 × 10^2^ CFU/mL), Lane 7 (*ST*-5.23 × 10^1^ CFU/mL, *SE*-3.59 × 10^1^ CFU/mL), Lane 8 (*ST*-5.23 × 10^0^ CFU/mL, *SE*-3.59 × 10^0^ CFU/mL), Lane 9 (NC). When the bacterial concentrations of *ST* and *SE* were below 10^1^ CFU/mL, the electrophoretic band was not visible under UV light. Therefore, the detection limits of gel electrophoresis for *ST* and *SE* in pure bacteria samples were 5.23×10^1^ and 3.59 × 10^1^ CFU/mL, respectively. *Salmonella typhimurium* (*ST*), *Salmonella enteritidis* (*SE*), negative control (NC), colony-forming unit (CFU).

To assess the detection limits of the dual RPA-LFD assay, a 10-fold serial dilution of the pure bacterial solution was prepared using purified bacterial genomic DNA as a template. The amplified products were analyzed by LFD and gel electrophoresis. When the bacterial concentrations of *S. typhimurium* and *S. enteritidis* were below 10^1^ CFU/mL, the color of the test line was not visible to the naked eye (Figure 4A). Therefore, the detection limits of the dual RPA-LFD assay for simultaneous detection of *S. typhimurium* and *S. enteritidis* were 5.23×10^1^ and 3.59 × 10^1^ CFU/mL, respectively.

**FIGURE 5:**
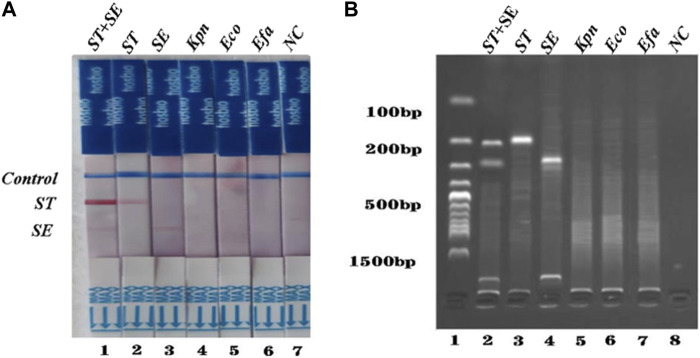
The specificity of dual RPA-LFD assay and gel electrophoresis for *ST* and *SE* in *7* pure bacteria samples. **(A)**. The specificity of dual RPA-LFD assay: *ST* and/or *SE* strain (Strip 1, 2, and 3) produced positive results, while other bacteria were negative: *Kpn* (Strip 4), *Eco* (Strip 5), *Efa* (Strip 6). **(B)**. The specificity of gel electrophoresis: *ST* and/or *SE* strain (Lane 1, 2, and 3) produced positive results, while other bacteria were negative: *Kpn* (Lane 4), *Eco* (Lane 5), *Efa* (Lane 6). *Salmonella typhimurium* (*ST*), *Salmonella enteritidis* (*SE*), *Klebsiella pneumoniae* (*Kpn*), *Escherichia coli* (*Eco*), *Enterococcus faecalis* (*Efa*).

To investigate the specificity of the dual RPA-LFD assay, DNA samples from a panel of *S. typhimurium*, *S. enteritidis,* and other common pathogens were extracted separately and used for the specificity determination of the dual RPA-LFD assay (Figure 5A strip 1, 2, 3, 4, 5, 6). Only *S. typhimurium* and *S. enteritidis* strains (Figure 5A stripe 1, 2, 3) tested positive, while the other bacteria including *Klebsiella pneumoniae* (stripe 4), *Escherichia coli* (stripe 5), *Enterococcus faecalis* (stripe 6) were tested as negative. The dual RPA-LFD assay was able to correctly differentiate *S. typhimurium, S. enteritidis,* and several other common pathogens, meaning that the dual RPA-LFD assay had a high degree of specificity. The experiment was repeated three times under the same conditions.

### 3.4 Application of the dual RPA-LFD assay in artificially contaminated samples

**FIGURE 6:**
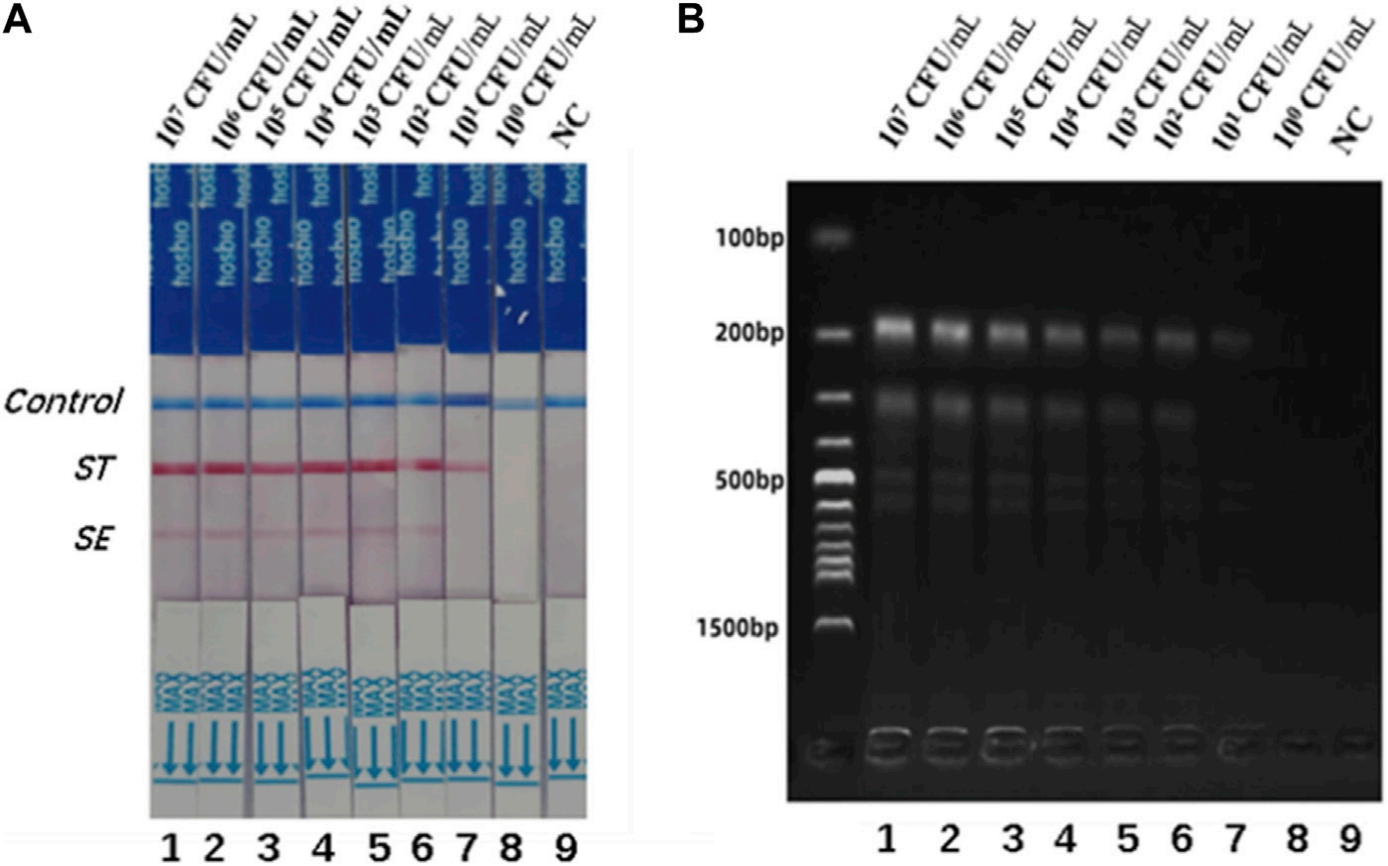
The detection limits of dual RPA-LFD assay and gel electrophoresis for *ST* and *SE* in artificially contaminated samples. **(A)**. The detection limit of dual RPA-LFD assay: Strip 1 (*ST*-8.30 × 10^7^ CFU/mL, *SE*-2.70 × 10^7^ CFU/mL), Strip 2 (*ST*-8.30 × 10^6^ CFU/mL, *SE*-2.70 × 10^6^ CFU/mL), Strip 3 (*ST*-8.30 × 10^5^ CFU/mL, *SE*-2.70 × 10^5^ CFU/mL), Strip 4 (*ST*-8.30 × 10^4^CFU/mL, *SE*-2.70 × 10^4^ CFU/mL), Strip 5 (*ST*-8.30 × 10^3^ CFU/mL, *SE*-2.70 × 10^3^ CFU/mL), Strip 6 (*ST*-8.30 × 10^2^ CFU/mL, *SE*-2.70 × 10^2^ CFU/mL), Strip 7 (*ST*-8.30 × 10^1^ CFU/mL, *SE*-2.70 × 10^1^ CFU/mL), Strip 8 (*ST*-8.30 × 10^0^ CFU/mL, *SE*-2.70 × 10^0^ CFU/mL), Strip 9 (NC). When the concentration of *ST* was below 10^1^ CFU/mL, and the concentration of *SE* was below 10^2^ CFU/mL, the color of the test line was not visible for the naked eye. Therefore, the detection limits of dual RPA-LFD assay for *ST* and *SE* in artificially contaminated samples were 8.30×10^1^ and 2.70 × 10^2^ CFU/mL, respectively. **(B)**. The detection limit of gel electrophoresis: Lane 1 (*ST*-8.30 × 10^7^ CFU/mL, *SE*-2.70 × 10^7^ CFU/mL), Lane 2 (*ST*-8.30 × 10^6^ CFU/mL, *SE*-2.70 × 10^6^ CFU/mL), Lane 3 (*ST*-8.30 × 10^5^ CFU/mL, *SE*-2.70 × 10^5^ CFU/mL), Lane 4 (*ST*-8.30 × 10^4^CFU/mL, *SE*-2.70 × 10^4^ CFU/mL), Lane 5 (*ST*-8.30 × 10^3^ CFU/mL, *SE*-2.70 × 10^3^ CFU/mL), Lane 6 (*ST*-8.30 × 10^2^ CFU/mL, *SE*-2.70 × 10^2^ CFU/mL), Lane 7 (*ST*-8.30 × 10^1^ CFU/mL, *SE*-2.70 × 10^1^ CFU/mL), Lane 8 (*ST*-8.30 × 10^0^ CFU/mL, *SE*-2.70 × 10^0^ CFU/mL), Lane 9 (NC). When the concentration of *ST* was below 10^1^ CFU/mL, and the concentration of *SE* was below 10^2^ CFU/mL, the electrophoretic band was not visible under UV light. Therefore, the detection limits of gel electrophoresis for *ST* and *SE* in artificially contaminated samples were 8.30×10^1^ and 2.70 × 10^2^ CFU/mL, respectively. *Salmonella typhimurium* (*ST*), *Salmonella enteritidis* (*SE*), negative control (NC), colony-forming unit (CFU).

Different concentrations of *S. typhimurium* and *S. enteritidis* were added to the negative stool sample to investigate the usefulness of the dual RPA-LFD assay. As shown in Figure 6, the detection limits of the dual LDF-RPA assay for *S. typhimurium* and *S. enteritidis* were 8.30×10^1^ and 2.70 × 10^2^ CFU/mL, respectively. The detection limits of the dual RPA-LFD assay for the detection of *S. typhimurium* in artificially contaminated samples were identical to those of the pure bacterial solution, but the detection limits for the detection of *S. enteritidis* in artificially contaminated samples were higher than those of the pure bacterial solution.

**FIGURE 7:**
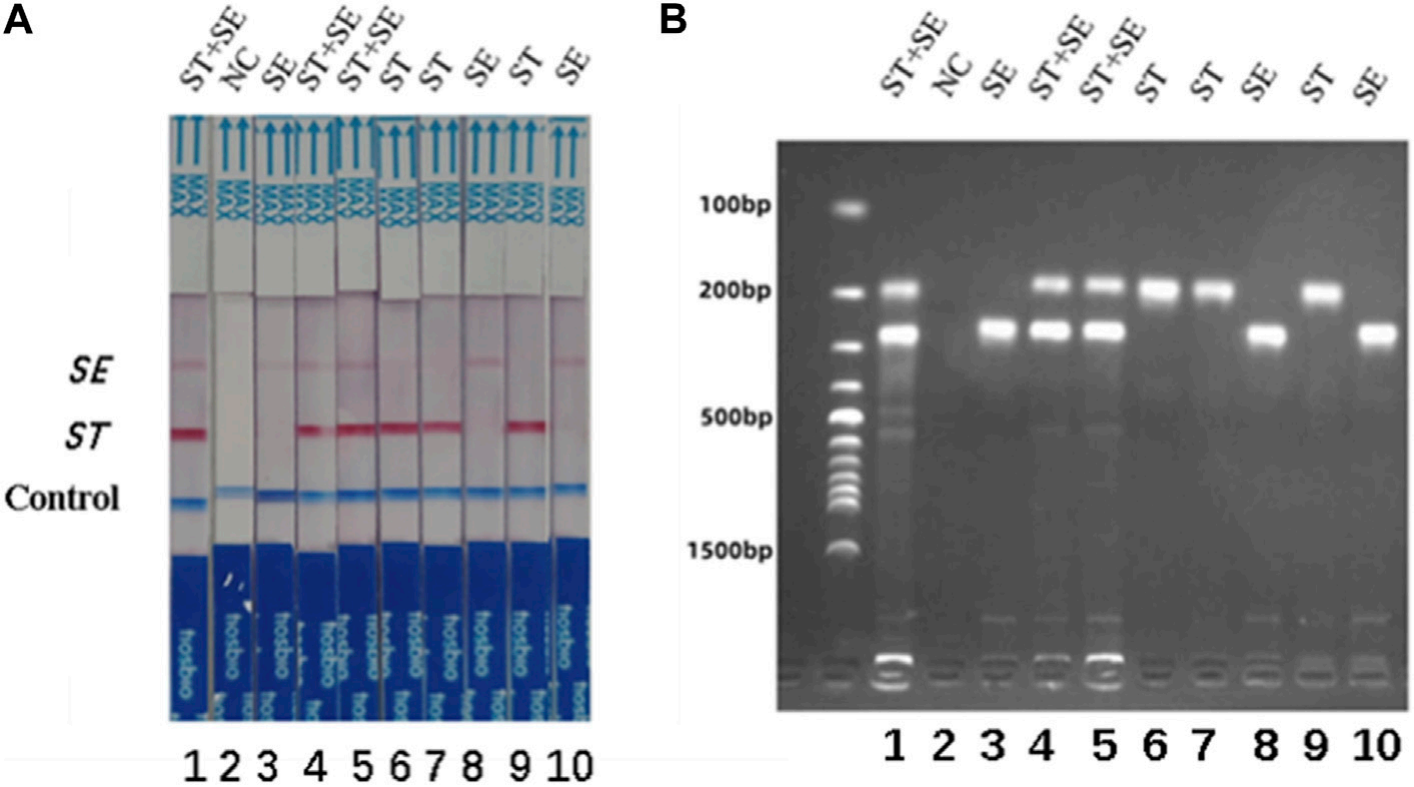
The specificity of dual RPA-LFD assay and gel electrophoresis for *ST* and *SE* in 10 artificially contaminated samples. **(A)**. The specificity of dual RPA-LFD assay: 3 samples were positive for *ST* and *SE* (Strip 1, 4, and 5), 3 samples were positive for *ST* (Strip 6, 7, and 9), 3 samples were positive for *SE* (Strip 3, 8, and 10), and 1 control was negative (Strip 2). The results above were completely consistent with the expectation. **(B).** The specificity of gel electrophoresis: 3 samples were positive for *ST* and *SE* (Lane 1, 4, and 5), 3 samples were positive for *ST* (Lane 6, 7, and 9), 3 samples were positive for *SE* (Lane 3, 8, and 10), and 1 control was negative (Lane 2). The results above were also consistent with the expectation. *Salmonella typhimurium* (*ST*), *Salmonella enteritidis* (*SE*).

The specificity of the dual RPA-LFD assay in artificial pollution samples were also investigated. In this experiment, a double-blind design was adopted. 10 unknown artificial contaminated samples were tested by dual RPA-LFD assay and the results were shown in Figure 7. 3 samples tested positive for *S. typhimurium* and *S. enteritidis*, 3 samples tested positive for *S. typhimurium*, 3 samples were tested positive for *S. enteritidis* and 1 control was tested as negative. The results were completely consistent with the actual contaminated samples.

### 3.5 Clinical sample testing

**FIGURE 8:**
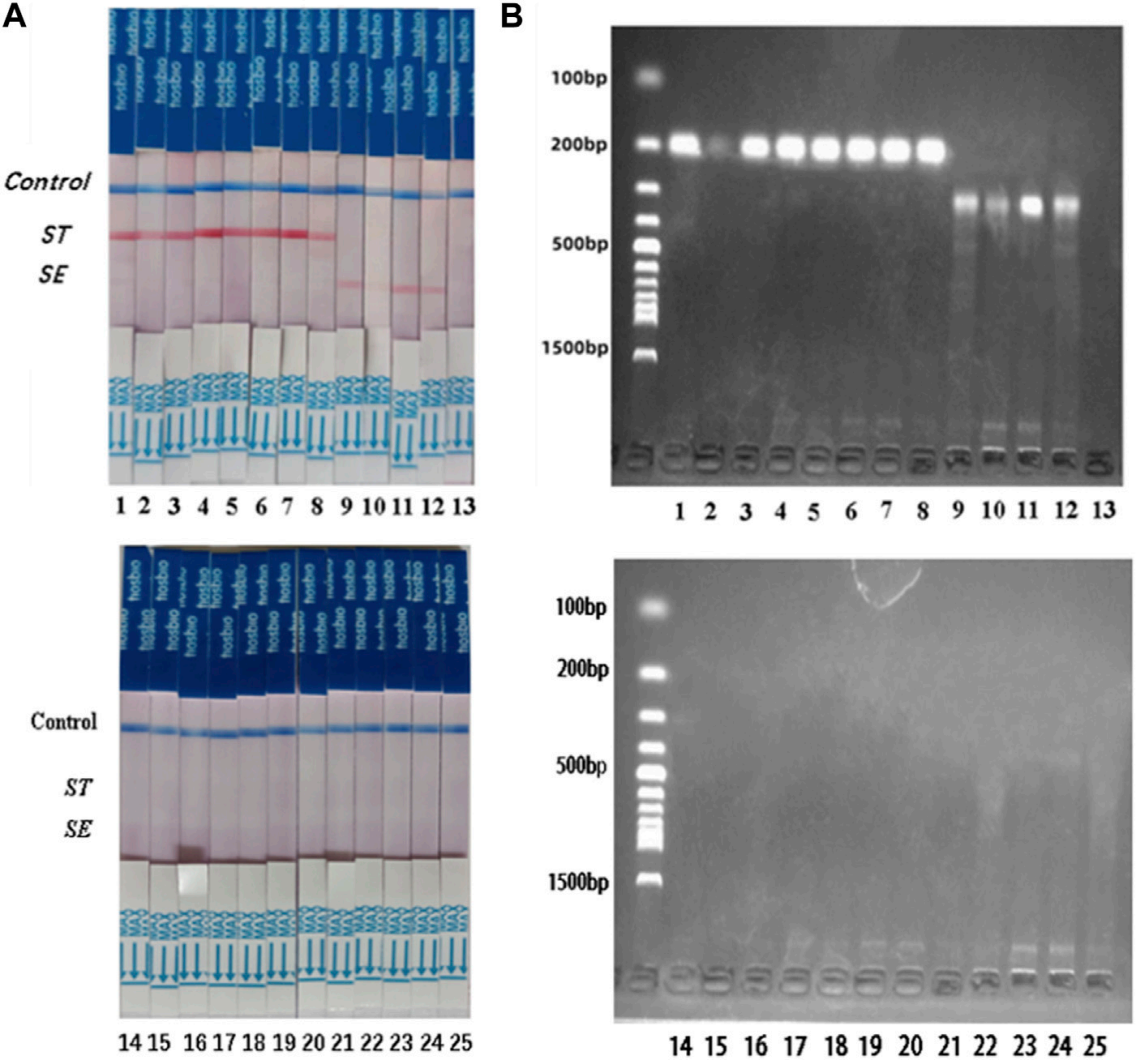
*ST* and *SE* detection by dual RPA-LFD assay and gel electrophoresis in 25 clinical samples. **(A)**. *ST* and *SE* detection by dual RPA-LFD assay: Strip 1, 2, 3, 4, 5, 6, 7, and 8 were positive for *ST*; Strip 9, 10, 11, and 12 were positive for *SE*; The rest strips were negative for *ST* and *SE*. The results above were completely consistent with the BAM method. **(B)**. *ST* and *SE* detection by gel electrophoresis: Lane 1, 2, 3, 4, 5, 6, 7, and 8 were positive for *ST*; Lane 9, 10, 11, and 12 were positive for *SE*; The rest strips were negative for *ST* and *SE*. The results above were also consistent with the BAM method. *Salmonella typhimurium* (*ST*), *Salmonella enteritidis* (*SE*), Bacterial Analysis Manual (BAM).

*S. typhimurium* and *S. enteritidis* were detected in 25 clinical samples using the dual RPA-LFD assay (Figure 8) and BAM. The results of the two tests were identical, with sensitivity and specificity of 100% (8/8) and 100% (17/17) for *S. typhimurium,* and 100% (4/4) and 100% (21/21) for *S. enteritidis* (Table 3).

**TABLE 3:** Comparison between dual RPA-LFD assay and BAM method.

Sample	*ST*		*SE*	
	Dual RPA-LFD assay	BAM	Dual RPA-LFD assay	BAM
1	+	+	-	-
2	+	+	-	-
3	+	+	-	-
4	+	+	-	-
5	+	+	-	-
6	+	+	-	-
7	+	+	-	-
8	+	+	-	-
9	-	-	+	+
10	-	-	+	+
11	-	-	+	+
12	-	-	+	+
13	-	-	-	-
14	-	-	-	-
15	-	-	-	-
16	-	-	-	-
17	-	-	-	-
18	-	-	-	-
19	-	-	-	-
20	-	-	-	-
21	-	-	-	-
22	-	-	-	-
23	-	-	-	-
24	-	-	-	-
25	-	-	-	-

*Salmonella typhimurium (ST), Salmonella enteritidis (SE),* Bacterial Analysis Manual (BAM).

## 4 Discussion

*S. typhimurium* and *S. enteritidis* were common foodborne pathogens that could cause a variety of foodborne illnesses, including typhoid fever, sepsis, gastroenteritis, and even death. Therefore, rapid, sensitive, and reliable detection of *S. typhimurium* and *S. enteritidis* was essential to reduce the risk factors caused by these two pathogens. There were many ways to detect pathogens in the stool, including real-time PCR (Sharma et al., 2023), enzyme-linked immunosorbent assay (Ghoshal et al., 2018), and electrochemical biosensors (Bacchu et al., 2022). However, these methods were not suitable for widespread application in field inspection due to the requirement of expensive instruments and time-consuming procedures. In recent years, with the emergence of isothermal amplification methods, such as nucleic acid sequence-based amplification (NASBA) (Compton, 1991), RPA (Piepenburg et al., 2006), and LAMP (Notomi et al., 2000), these problems have been partly overcome.

However, more and more patients were infected with multiple pathogens, and traditional single-target nucleic acid detection could not meet the needs of multi-pathogen detection. Therefore, we needed to develop a multi-nucleic acid assay that could achieve rapid amplification of multiple pathogens simultaneously in a single reaction system. This approach not only maintained the specificity and sensitivity of single-target nucleic acid detection but also reduced the number of procedures and reagents (Santiago-Felipe et al., 2015). Multiplex PCR and multiplex RPA were two widely used multiplex nucleic acid detection techniques. Both multiplex PCR and multiplex RPA have been used to detect a variety of foodborne pathogens. For example, multiplex PCR was used for the simultaneous detection of herpes simplex virus-1, herpes simplex virus-2, varicella-zoster virus, Epstein-Barr virus, and cytomegalovirus in cerebrospinal fluid (Golrokh Mofrad et al., 2022), and multiplex RPA was used for the triple detection of three foodborne pathogens in seafood (Ma et al., 2020).

Nevertheless, the reliance on the thermal cyclers limited the application of PCR, whereas RPA had significant advantages such as short amplification time, low temperature, and high tolerance to sample impurities. However, reports of simultaneous detection of two or more foodborne pathogens by the RPA-LFD assay were rare (Lobato and O'Sullivan, 2018). In this study, we described a specific dual RPA-LFD assay for the simultaneous and accurate detection of *S. typhimurium* and *S. enteritidis*. The dual RPA-LFD assay could be performed at constant temperatures without the need for specialized instrumentation. Therefore, this method improved the ability to perform multiplex nucleic acid amplification outside the laboratory.

In a dual RPA reaction, amplification efficiency was determined by the selection of target sequences and primer design (Lobato and O'Sullivan, 2018). The primers for dual RPA were designed for the STM4497 gene of *S. typhimurium* and the safA gene of *S. enteritidis*. The STM4497 gene was specific to *S. typhimurium* and had high accuracy in identifying *S. typhimurium* (Kim et al., 2006). The safA gene was specific to *S. enteritidis* and had little homology with other known prokaryotes in the GenBank database (Lauri et al., 2011). However, it is important to note that in dual RPA, the amplification of one target may impact the amplification of the other target (Jauset-Rubio et al., 2018), Therefore, we adjusted the primer concentration of *S. typhimurium* and *S. enteritidis* to a 1:1 ratio to ensure the comparable amplification efficiency of both fragments. The performance of RPA could be affected by several factors such as reaction temperature, reaction time, and primer concentration. The amplification efficiency could be affected by the reaction temperature through its impact on enzyme activity in the system. Moreover, the reaction time showed a positive correlation with the number of cycles, excessive cycles could result in non-specific amplification while insufficient cycles could reduce amplification efficiency. Furthermore, primer concentration significantly affected RPA efficiency, over-high concentration could lead to non-specific amplification while over-low concentration could also decrease amplification efficiency. To minimize these influences in this experiment for dual RPA detection of *S. typhimurium* and *S. enteritidis*, optimization was performed on the reaction temperature, reaction time, and primer concentration. The optimal conditions for double RPA detection of *S. typhimurium* and *Salmonella* enteritidis were achieved at 38 °C for 20 min with a primer concentration ratio of 1:1. Previous studies have shown that RPA reactions could be carried out at ambient temperatures, and even at body temperature (Lillis et al., 2014; Wang et al., 2017).

In recent years, an increasing number of detection methods have been combined with RPA, including polymer flocculation sedimentation (Hu et al., 2020), and surface-enhanced Raman scattering (Jianwen et al., 2022). Compared with these detection methods, LFD was a faster and simpler detection method. It could get visual results in 5–10 min. To achieve double detection, we labeled the 5′end of each upstream primer with biotin and FAM. All downstream primers were labeled with digoxin at the 5′end. Two double-labeled products were formed during amplification. At the same time, an LFD with two test lines was prepared for the detection of dual RPA amplification products. The detection limits of *S. typhimurium* and *S. enteritidis* in pure culture samples were determined to be 5.23 × 10^1^ CFU/mL and 3.59 × 10^1^ CFU/mL, respectively. However, the detection limits of *S. typhimurium* and *S. enteritidis* in artificially contaminated samples were found to be 8.30 × 10^1^ CFU/mL and 2.70 × 10^2^ CFU/mL, respectively. The detection limits of *S. typhimurium* and *S. enteritidis* in artificially contaminated samples were higher than those in pure culture samples, which might be attributed to the inherent complex composition of artificially contaminated samples. In addition, the detection limits of *S. typhimurium* and *S. enteritidis* in the same sample were also different, which might be attributed to different amplification efficiency of two target genes in the same reaction system. The sensitivity of this assay could also be affected by some external factors, such as the time of infection of *S. typhimurium* and *S. enteritidis*, whether to use drugs and differentiated immunity of different patients. In addition, there was no cross-amplification not only between the two target bacteria but also with other bacteria. The result showed that the proposed method was highly specific.

Herein, the dual RPA-LFD assay established in this study was further compared with the methods in other studies. As shown in Table 4, Compared to the RAA-Microfluidic Chip, RPA-LFD, RT-LAMP, Colony and bacterial LAMP, Digital PCR, and CRISPR/Cas-based biosensors methods, the dual RPA-LFD assay was able to detect a lower concentration of *S. typhimurium* and *S. enteritidis* DNA and able to achieve rapid amplification of two pathogens simultaneously in a single reaction system, this approach reduced the number of procedures and reagents. In addition, the sensitivity and specificity of dual RPA-LFD assay for detecting *S. typhimurium* and *S. enteritidis* were preliminarily verified using clinical samples in this study. Regarding detection time, the Colony and bacterial LAMP method did not require DNA extraction, resulting in shorter detection times compared to RT-LAMP (Techathuvanan et al., 2010; Zhong et al., 2024). Detection of *S. enteritidis* by RT-LAMP and PCR required bacterial enrichment, leading to a longer detection time (Guo et al., 2000; Techathuvanan and D'Souza, 2012). However, the dual RPA-LFD assay did not require thermal cycling or bacterial enrichment, allowing for a detection result within 65 min. The use of dual RPA-LFD strips was beneficial to immediate detection. However, prolonged exposure to air could compromise the stability of dual RPA-LFD strips, thereby increasing the risk of false positive results compared to fluorescence tests (Hou et al., 2022). Moreover, the interaction between two sets of primers for *S. typhimurium* and *S. enteritidis* in dual RPA reaction posed a big challenge for primer design. For LAMP assays, fluorescence or turbidimetry was typically employed for visual readout results. However, it could be challenging to identify color changes when detecting low-concentration samples. In our study, the LFD strips used to detect fluorescence signals were suitable to be applied for point-of-care testing and in the laboratories of resource-poor areas.

**TABLE 4:** Comparison between the present study and previous detection methods for *ST* and *SE*.

ID	Method	Limit of detection	Specificity	Sensitivity	detection time (min)	Visual detection	ref.
1	RAA-Microfluidic Chip	89 CFU/mL	-	-	40	No	Wu et al. (2022)
2	RPA-LFD	1.9 CFU/mL	-	-	10	Yes	Hu et al. (2019)
3	RT-LAMP	10^2^ CFU/25 g	-	-	90	Yes	Techathuvanan et al. (2010)
4	Colony and bacterial LAMP	10^2^ CFU/reaction	-	-	60	No	Zhong et al. (2024)
5	Digital PCR	90 CFU/reaction	-	-	120	No	Suo et al. (2022)
6	CRISPR/Cas-based biosensors	7.9 × 10^1^ CFU/mL	-	-	120	No	Duan et al. (2023)
7	PCR	10^1^ CFU/mL	-	-	400	Yes	GUO et al. (2000)
8	RT-LAMP	10^7^ CFU/25 mL	-	-	1,440	Yes	Techathuvanan and D'Souza (2012)
9	dual RPA-LFD assay	8.30 × 10^1^ CFU/mL	100%	100%	65	Yes	This study
10	dual RPA-LFD assay	2.70 × 10^2^ CFU/mL	100%	100%	65	Yes	This study

*Salmonella typhimurium (ST), Salmonella enteritidis (SE),* colony-forming unit (CFU).

To more fully confirm the validity and applicability of sample analysis using dual RPA-LFD assay, artificially contaminated samples and clinical samples were analyzed and compared with microbial cultures. The result showed that the dual RPA-LFD assay had high sensitivity and specificity in both artificially contaminated samples and clinical samples. In conclusion, dual RPA-LFD assay was a fast, sensitive, and high-throughput method for the detection of foodborne pathogens.

## 5 Conclusion

In this study, a dual RPA-LFD assay was developed for the rapid and simultaneous detection of two food-borne pathogens *S. typhimurium* and *S. enteritidis* in the stool. The dual RPA-LFD assay was time-saving, simple, sensitive, and specific, allowing visual analysis of *S. typhimurium* and *S. enteritidis* in a single reaction. The entire reaction process, including amplification and result reading, could be completed within 65 min. The detection limits of *S. typhimurium* and *S. enteritidis* in pure culture samples were 5.23 × 10^1^ CFU/mL and 3.59 × 10^1^ CFU/mL, respectively. The detection limits of *S. typhimurium* and *S. enteritidis* in artificially contaminated samples were 8.30 × 10^1^ CFU/mL and 2.70 × 10^2^ CFU/mL, respectively. In addition, this assay had potential application outdoors, as it could be performed within 1 h at 38°C without a complex instrument, and the results could be observed with the naked eye. In conclusion, the dual RPA-LFD assay established in this study had practical significance for the rapid detection of *S. typhimurium* and *S. enteritidis* in the future.

## Data Availability

The original contributions presented in the study are included in the article/Supplementary material, further inquiries can be directed to the corresponding authors.
